# Supplementary material for: Multi-scale ResNet and BiGRU automatic sleep staging based on attention mechanism
Source: PLoS One. 2022 Jun 16;17(6):e0269500. doi: 10.1371/journal.pone.0269500 (PMC9202858; doi:10.1371/journal.pone.0269500)
Supplement: Attachment — Submitted filename: Response to Reviewers.docx [file pone.0269500.s002.docx]

Dear Editor and Reviewers,

We are very glad to hear from you, we are truly grateful to yours and other reviewers’ comments and thoughtful suggestions on our manuscript entitled “Multi-scale ResNet and BiGRU automatic sleep staging based on attention mechanism”. Those comments are all valuable and very helpful for revising and improving our paper, as well as the important guiding significance to our researches. We have carefully studied the comments and suggestions made by the reviewers and have made corrections. Please review again. The revised part is marked in red in the paper.

The research grant of this project is stated as follows:

This project is funded by the National Natural Science Foundation of China (51779050) and the National Natural Science Foundation of Heilongjiang Province (F2016022). All the funds obtained for this study were used to purchase corresponding experimental equipment and conduct experimental tests, and the researcher did not receive salary from the sponsors. The funders had no role in study design, data collection and analysis, decision to publish, or preparation of the manuscript.

The main corrections in the paper and the responds to the reviewer’s comments are as following:

**Response to first reviewer’s comments**

1. **Comment:**

The literature of the paper is poor, Recommended to provide the suitable literature by considering the recently published papers.

Response:

Thanks for reviewer’s comment. The author has added 7 literatures on the original basis according to the comments of reviewer’s, and the positions in the paper are [27] to [30] and [33] to [35].

2. **Comment:**

Some of the formulae of paper are available in the existing published papers.

**Response:**

Thanks for reviewer’s comment. The author has added the corresponding formula in the theoretical analysis of bidirectional gated loop unit network, see formula (6), from lines168 to 172.

3. **Comment:**

There are several performance metrics in the literature, but the authors do not consider more. It is recommended to perform the experiments using multiple datasets and metrcs.

**Response:**

Thanks for reviewer’s comment. The author added three evaluation indexes, including loss function, recall rate and classification recognition rate of total sleep cycle, to evaluate the classification results in the manuscript, as shown in Fig. 8, Fig. 9 and Fig. 10. This part is conducted from 455 lines to 498 lines in the evaluation / classification experiment of the manuscript.

The author added two data sets UCD and SHHS to conduct the experiment according to the opinions of reviewer’s, as shown in Table 2 and Table 3. The content is in the part of data sources and preprocessing，evaluation/classification experiment of the manuscript, from lines 313 to 320 and 403 to 420.

4. **Comment:**

  The reasons for achieving the superior performance to be listed. also list the limitations.

**Response:**

Thanks for reviewer’s comment. Reasons for the excellence of the author's research: The model proposed by the author uses a variety of convolution kernels of different sizes for feature extraction of sleep signals, so that the intrinsic sleep information of EEG signals can be fully mined. Secondly, the bidirectional gating loop unit network is introduced to analyze time sequence information in detail aiming at the problems of limited learning ability of sleep cycle features caused by the inability of traditional algorithms to recognize time sequence patterns in long-term associated data.

Limitations of the authors' study: the sources of physiological signals used in the experiment are limited.

**Response to second reviewer’s comments**

1. **Comment:**

Abstract could be little more precise and crisp.

**Response:**

Thanks for your advice. The abstract has been revised as suggested by the reviewer’s.

2. **Comment:**

What audience would benefit most from this work?

**Response:**

Thanks for reviewer’s comment. People with acute diseases in sleeping and those who do automatic sleep staging studies can benefit the most.

3. **Comment:**

What do you see as this manuscript’s main contribution to the literature in the Automated Sleep staging? Summarize in a few sentences.

**Response：**

Thanks for reviewer’s comment. The main contribution is to provide a new method for the study of automatic sleep staging, and compared the method proposed in this thesis with the method proposed by previous researchers, highlighting the advantages of this study. The model proposed in this paper has a higher recognition rate and better biological interpretability. The feasibility and validity of automatic sleep staging using residual network and bidirectional gating cycle network are proved by experiments.

**4. Comment:**

What are the three strongest aspects of this manuscript?

**Response：**

Thanks for reviewer’s comment. The first aspect: In view of the limited learning ability of sleep cycle characteristics caused by the inability of traditional algorithms to recognize time series patterns in long-term associated data, Bidirectional Gated Recurrent Unit (BiGRU) is introduced to comprehensively and detail analyze time series of information.

The second aspect: Multi-scale learning method is proposed to excavate the sleep characteristics of EEG sleep signals more fully.

The third aspect: In this thesis, the improved algorithm model MARnet-BigRU is tested on Seep-EDF, UCD and SHHS data sets, and the recognition rate reaches 84.24%, 79.34% and 81.60% respectively. The sleeping classification recognition rate is better than other models and the recognition rate improved by 22.04%, 19.64% and 21.36% compared with the original residual model. It has better generalization ability and robustness.

**5. Comment:**

I would like to encourage authors to include some more discussions and reviews of latest research works and existing techniques.

**Response:**

Thanks for reviewer’s comment. The authors have discussed and commented on the latest research and existing technologies in lines from 76 to 90 of the introduction.

**6. Comment:**

Any hyperparameter selection method employed?

**Response:**

Thanks for reviewer’s comment. The selection of hyperparametric is made by the author through the setting of different parameters. Then a large number of comparative experiments are carried out to select the results, and the experiment results proves that the choice of this study is the most appropriate.

**7. Comment:**

In the introduction of the article, the study innovation should be clearly specified.

**Response:**

Thanks for reviewer’s comment. The author has listed the innovations of the research in the introduction of the article, and there are three innovations in total at lines from 96 to 108.

We did our best to improve the manuscript and made some revisions to it. We hope that these amendments are satisfactory.

Many thanks to the editors and reviewer’s for their work on our paper.

Thank you again for your advice and suggestions.

Wish you all the best!

Sincerely yours,

Changyuan Liu, Multi-scale ResNet and BiGRU automatic sleep staging based on attention mechanism

No.52 Xuefu Road, Harbin City, Heilongjiang Province, China,18646103995, liuchangyuan@hrbust.edu.cn
